# Comparison of automated algorithm-based versus manual dental landmark placement for model analysis and evaluation of orthodontic quality indices

**DOI:** 10.1093/ejo/cjag063

**Published:** 2026-08-26

**Authors:** Norbert Alexander Lang, Franziska Alina Lang, Christian Niederau, Anne Schieberle, Kay Stankov, Michael Wolf, Rogerio Bastos Craveiro

**Affiliations:** Department of Orthodontics, University Hospital RWTH Aachen, Pauwelsstraße 30, 52074 Aachen, Germany; Department of Orthodontics, University Hospital RWTH Aachen, Pauwelsstraße 30, 52074 Aachen, Germany; Department of Orthodontics, University Hospital RWTH Aachen, Pauwelsstraße 30, 52074 Aachen, Germany; Department of Orthodontics, University Hospital RWTH Aachen, Pauwelsstraße 30, 52074 Aachen, Germany; Stat4med, Große Seestraße 44, 60486 Frankfurt am Main, Germany; Department of Orthodontics, University Hospital RWTH Aachen, Pauwelsstraße 30, 52074 Aachen, Germany; Department of Orthodontics, University Hospital RWTH Aachen, Pauwelsstraße 30, 52074 Aachen, Germany

**Keywords:** dental landmark placement, orthodontic quality index, PAR index, ABO eCRE, semi-automated algorithm, digital model analysis

## Abstract

**Background:**

Digital approaches have fundamentally transformed orthodontics. This study compares an automated, rule-based algorithm with manual landmark placement, evaluating the results of manual versus semi-automated orthodontic quality indices.

**Methodology:**

This retrospective method-comparison study evaluated 100 digital models from 50 orthodontic patients using the Onyxceph3™ diagnostic software. Primary automated landmark placement was assessed for four predefined dental landmark types across 14 tooth groups and was then compared with expert-based manual landmark placement, which was used as the reference standard. Deviations were quantified using Euclidean distances and signed coordinate differences along the X-, Y- and Z-axes. Secondly, we evaluated semi-automated versus manual orthodontic quality assessment using the ABO eCRE and PAR index.

**Results:**

Cusps and incisal landmarks show low levels of error when using automatic placement compared with manual placement, with a threshold of ≤1 mm. Vestibular marginal gingival landmarks show the highest deviation. Across tooth groups, mean deviations ranged from 0.54 mm for second molars (95% CI 0.49–0.59) to 0.83 mm for canines (95% CI 0.80–0.85). There was no relevant global directional bias in the X-, Y- and Z-axes. Secondary analysis revealed mean differences of 1.90 points and 3.98 points for ABO eCRE, and −0.08 points and 0.74 for PAR in initial and final models, respectively.

**Conclusion:**

The automatic placement of landmarks generally showed acceptable deviations, with the exception of vestibular marginal gingival landmarks. However, the automatic detection of landmarks and orthodontic indices should always be reviewed by a clinician.

## Introduction

The development of digital approaches has fundamentally changed the field of orthodontics, particularly regarding to diagnosis, assessment, and treatment. These approaches present a variety of opportunities for advancing research [1]. Increasing digitalization enables efficient storage and exchange of data, making standardized, reproducible and efficient measurement methods possible [2–4]. The very frequent use and evaluation of digital models, not only in orthodontics but in all areas of dentistry, makes this a highly sought-after topic not only in research but also in clinical practice. This process supports and improves the quality of documentation, diagnosis and treatment, which greatly improves the patient experience [2, 5–7]. Consequently, there is a growing interest in approaches that reduce the workload for examiners while ensuring reliable, clinically meaningful and reliable measurements [7, 8]. Manual landmark placement remains operator-dependent and may limit the standardization of digital model analyses. The current trend is towards using validated digital software-based measurement tools. Through more standardized and reproducible landmark placement, automated methods can facilitate the efficient calculation of orthodontic indices and support broader implementation of index-based assessment in digital workflows. Using indices more consistently in clinical practice can strengthen the evaluation of treatments and the monitoring of quality and contribute to optimizing individual treatment strategies [3]. Therefore, automated landmarks offer a promising solution to this challenge [3, 7, 9]. The landmarks allow for the assessment of orthodontic treatment to be made using orthodontic indices, which are used to measure the effectiveness of treatment.

Orthodontic indices such as the Peer Assessment Rating (PAR) index [10] and the American Board of Orthodontics Cast–Radiograph Evaluation (ABO-CRE) [6] score play a central role in clinical decision-making, treatment evaluation, quality assurance and research. These indices enable objective comparison of treatment outcomes [6, 10]. However, their calculation is based on the precise identification of landmarks, which traditionally must be set manually by trained examiners. It is necessary to establish whether automated landmark detection can deliver consistent and reliable measurements. Demonstrating that automated landmark placement is not inferior to the manual gold standard is therefore an important step towards achieving a fully automated and standardized assessment process within digital orthodontic workflows.

Our primary objective is to compare the absolute deviation of automated, algorithm-based landmark placement with manual landmark placement as a reference within tooth groups in digital orthodontic patient models. Secondly, we investigate whether potential deviations in automated landmark identification could affect the calculation of PAR and ABO-CRE scores. We hypothesize that, compared with manual reference measurements, automated landmark placement would lead to comparable and acceptable results, enabling reliable, index-based assessments without influencing the index values in a clinically relevant way.

## Materials and methods

### Study design

**Figure 1:**
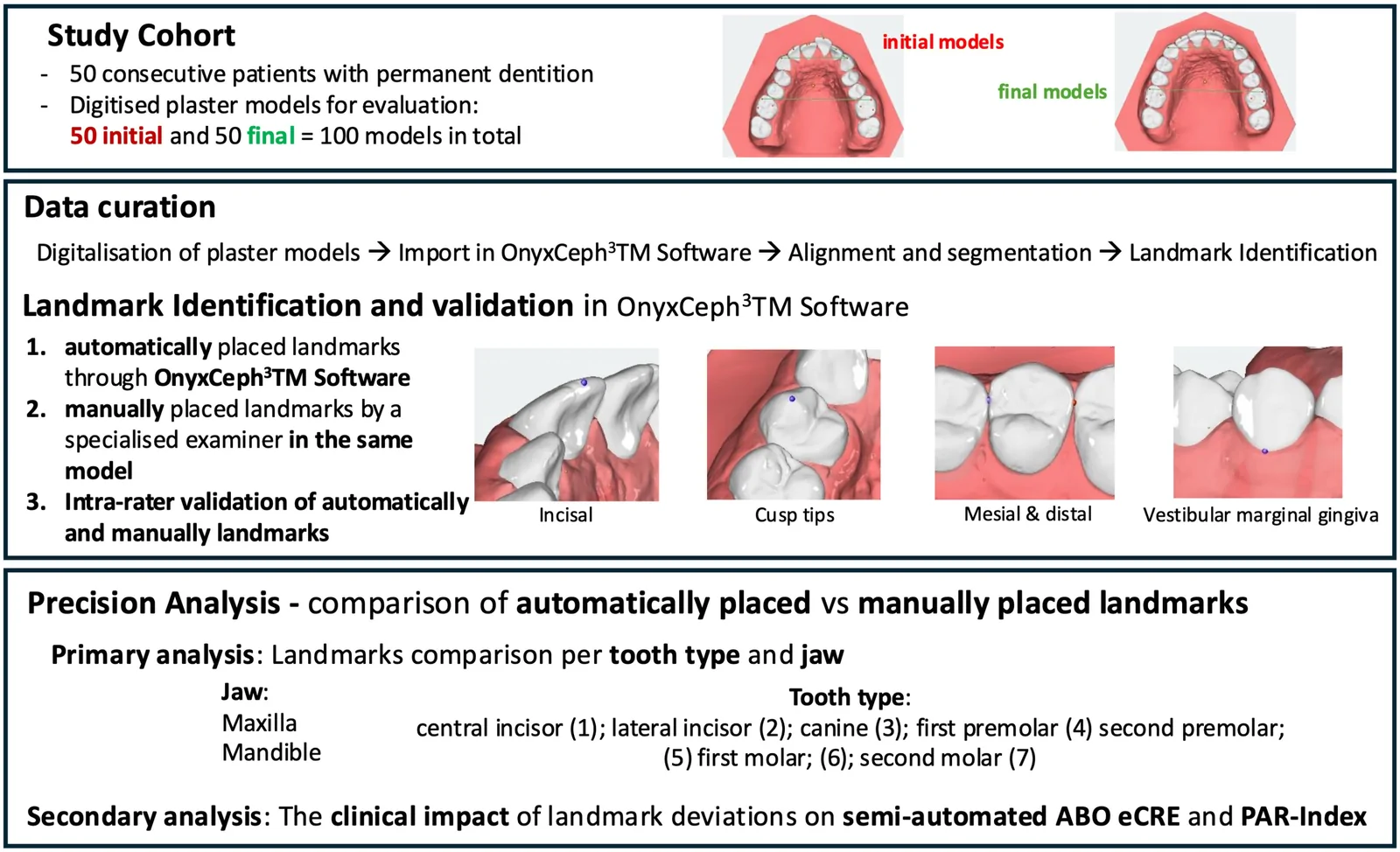
An overview of the study design, including a description of study cohort, data curation, landmark identification and the analysis of landmark placement.

This retrospective study examined the accuracy of automated compared with manual landmark identification on digital dental models, as well as its effect on index-based orthodontic assessment scores (Fig. 1). This study is in accordance with the STROBE guidelines for cohort studies (Supplementary Table S1). Ethical approval was obtained from the ethics committee of Medical Faculty of RWTH Aachen with the ethical registration number EK 25-302.

### Participants, eligibility criteria, and setting

This present study was conducted using digital dental models from orthodontic patients treated at the University Hospital RWTH Aachenbetween April 2019 and January 2026. The analysis involved 50 initial models taken before the start of orthodontic treatment and 50 final models taken after completion of treatment for the same patient. For both manual and automatic landmark analysis, as well as for index evaluation, identical segmentation, separation and completion of the tooth crowns for each model were applied to ensure an identical measurement baseline. Automated and manual landmark placement, coordinate-based deviation analyses and subsequent PAR and ABO eCRE score calculations were performed retrospectively on the stored digital models.

The inclusion criteria were as follows: (1) fully erupted permanent dentition except for the third molars; (2) sufficient scan quality; (3) irrespective of crowding, malocclusion, previous orthodontic treatment or prosthetic restorations and fillings. The exclusion criteria were: (1) craniofacial anomalies or syndromes.

The eligibility criteria were defined so that the study cohort would reflect routine orthodontic patients encountered in daily clinical practice. Of the cases initially screened, two patients were excluded due to insufficient scan quality, which prevented the reliable identification of landmarks. Following the final case selection, there were no further exclusions.

### Data acquisition

The scans were performed using an intraoral scanner (Medit i600, Medit Corp., Seoul, South Korea) and exported as STL files. These were imported into the orthodontic diagnostic software OnyxCeph^3^™ (Image Instruments GmbH, Chemnitz, Germany; version 3.2.240) and processed using the software’s segmentation workflow. The segmentation procedure included the steps crown segmentation, crown separation, crown completion and landmark checking. This workflow identifies areas of the closed scan surface belonging to individual tooth crowns and converts them into separate completed 3D tooth objects. Missing surface information not captured by the scanner, such as contact areas and root-related extensions, is supplemented by the software. This procedure generated a patient-specific three-dimensional coordinate system for each model. In the oriented digital models, these X-, Y- and Z-axes corresponded to the transverse, vertical and sagittal directions, respectively.

Automated landmark detection was then carried out using the deterministic, rule-based OnyxCeph^3^™ algorithm to identify specific dental landmarks (incisal, cusp tip, mesial and distal point, and vestibular marginal gingiva). In addition, all remaining manual landmarks required for the assessment of the semi-automated PAR and ABO electronic Cast–Radiograph Evaluation (eCRE) were supplemented. The *x*, *y* and *z* coordinate of all landmarks, as well as the index values, were exported and saved. Manual landmark placement was performed on cloned segmented models after removal of the automatically generated landmarks. Discrepancies were resolved jointly according to the predefined anatomical landmark definitions. The agreed final landmark positions were implemented by the primary examiner and used as the expert-based reference standard for comparison with automated landmark placement.

The vector distance (*V*) in millimetres (mm) from the model-specific coordinate origin to the manually (*M*) and automatically (*A*) defined landmark coordinates was determined using the following formula: $|V|=\sqrt{x^{2}+y^{2}+z^{2}}$

The difference vector (automatic minus manual) was calculated for each landmark using the following Euclidian distance to quantify the three-dimensional spatial deviation (mm):


$$|D|=\sqrt{{\left(x_{A}-x_{M}\right)}^{2}+{\left(y_{A}-y_{M}\right)}^{2}+{\left(z_{A}-z_{M}\right)}^{2}}$$

### Examiner calibration and consensus-reaching

Manual landmark placement was performed by two orthodontic specialists with several years of clinical experience and specific expertise in digital orthodontic measurements. Before data collection, both examiners were calibrated using digital dental models that were not included in the final study sample. The calibration process included a joint review of the predefined landmark definitions, anatomical criteria and the standardized landmark placement procedure in OnyxCeph^3^™. The examiners were blinded to the automated landmark coordinates and index results during manual placement and consensus review. Discrepancies were resolved jointly according to the predefined anatomical landmark definitions. The agreed final landmark positions were implemented by the primary examiner and used as the expert-based reference standard for comparison with automated landmark placement.

### Landmarks, tooth type and semi-automated orthodontic indices

The landmarks were as follows: (1) cusp tip landmark: centre of the cusp; (2) mesial and distal landmark: the mesial and distal points of the largest mesial-distal crown diameter [11]; (3) incisal landmark: central point on the incisal edge and (4) vestibular marginal gingival landmark: most apical point of the vestibular marginal gingival contour. The deviation was aggregated by arithmetic means per tooth type, separately for the maxillary and mandibular dentitions, and for the following tooth group: Central incisor (1); lateral incisor (2); Canine (3); First premolar (4) Second premolar; (5) First molar; (6); Second molar (7).

In a semi-automated workflow, the software performs most of the landmark detection and measurement calculations automatically, while the user must perform certain steps of the analysis manually. The semi-automated ABO eCRE was developed in collaboration between RWTH Aachen University Hospital and the OnyxCeph^3^™ development team and is expected to be available by the end of 2026. A total of 66 automatic and 73 manual landmarks must be set. The following subsections were defined and analysed: maxilla and mandible alignment; marginal ridges; buccolingual inclination of the upper and lower jaw; overjet 3–3 and 4–7; and occlusal relationship. A total of 38 automatic and 8 manual landmarks must be set for the semi-automated PAR index [3]. The subsections are as follows: upper and lower anterior segment, buccal occlusion, overbite and midline.

### Outcomes

The primary outcome was the absolute three-dimensional geometric deviation between automated and expert-based manual landmark placement, expressed as Euclidean distance in millimetres. The measurement method—automated versus manual assessment—was the comparison factor, with manual expert-based landmark placement serving as the reference standard. Secondary outcomes were the signed coordinate differences along the X-, Y-, and Z-axes and the paired differences in PAR and ABO eCRE scores between semi-automated and manual assessment. Model stage, jaw, tooth group, and landmark category were prespecified as potential sources of measurement variability and were examined in stratified analyses. Because both assessment methods were applied to the same digital models and no causal exposure–outcome association was investigated, no confounders in the aetiological sense were prespecified and no confounder-adjusted analysis was performed.

### Sources of Bias and comparability between exposure groups

Various measures were taken to minimize potential sources of bias. Both automatic and manual landmark placement were carried out on identical digital models, using the same segmentation and three-dimensional coordinate system, to ensure direct methodological comparability.

To avoid influencing manual identification with automatic landmark placement, all automatically generated landmarks were removed prior to manual placement. Manual identification was carried out by two trained assessors. Intrarater and interrater reliability were used to assess the consistency of the reference measurements. Scan quality was considered a potential of variation in the measurements. To minimize measurement bias, digital models with insufficient scan quality were excluded according to predefined suitability criteria.

### Reliability

Reliability of manual and automated landmark placement was assessed using intraclass correlation coefficients (ICCs). For intrarater reliability, 10 initial models were randomly selected and re-evaluated by the primary examiner after a 3-month interval. For the algorithm-based workflow, segmentation was repeated before the second measurement to include segmentation as a clinically relevant source of variability. As the deterministic algorithm is expected to produce the same coordinates for an unchanged segmentation, this approach enabled the evaluation of the entire automated workflow, including segmentation, as a clinically relevant source of variability. Interrater reliability of manual landmark placement was assessed using a randomly selected subset of 10 initial digital models, which were independently landmarked by both examiners before consensus adjustment. ICCs were calculated using a two-way model for absolute agreement based on single measurements. For intrarater reliability, ICCs were calculated between the first and second measurements; for interrater reliability, ICCs were calculated between the two examiners. All ICCs were computed in R for all predefined spatial dimensions (X, Y and Z).

### Sample size calculation

The sample-size calculation was based on the primary landmark-deviation outcome and on the patient as the independent analysis unit. The statistical hypothesis was that the patient-level mean absolute Euclidean landmark deviation would be below the clinically acceptable threshold of 1.0 mm. Assuming an expected mean deviation of 0.55 mm and a standard deviation of 0.49 mm [12], ∼8 independent patients were required at a one-sided significance level of *α* = 0.05 and 80% power (1 − *β* = 0.80). For 90% power (1 − *β* = 0.90), ∼11 patients would be required. A total of 50 eligible patients were included, exceeding the calculated minimum for the primary outcome. Because no reliable prior estimates of the within-patient standard deviation of paired ABO eCRE and PAR score differences were available, no separate formal sample-size calculation was performed for these secondary noninferiority analyses.

### Statistical analysis

Statistical analyses were performed using R (version 4.5.2; R Foundation for Statistical Computing, Vienna, Austria). For the dental landmark analysis, the absolute deviation between automatic and manual dental landmark positions was calculated in millimetres and additionally classified into clinically interpretable categories defined *a priori* as ideal (≤ 0.5 mm), moderate (> 0.5 to 1 mm), adverse (> 1 to 2 mm), and severe (> 2 mm). In addition to descriptive statistics, this categorical approach was used to facilitate the clinical interpretation of geometric agreement between methods across landmark groups. Coordinate-wise differences were used as an exploratory analysis to assess whether automated landmark placement showed a systematic directional displacement relative to manual expert placement.

For the secondary noninferiority analyses, semi-automated ABO eCRE and PAR scores were compared with the corresponding manual scores. For the quality scores ABO and PAR, noninferiority was evaluated using a margin of 5 points for the total score and 1 point for each subsection. For the total ABO eCRE score, a maximum difference of 5 points was defined, based on previous studies of the ABO Model Grading System in which a difference of 5 points in the mean total score was considered to be clinically significant or meaningful [13, 14]. For individual subsections, the maximum difference was defined as 1 point, since the ABO scoring system uses discrete penalty-point categories, with one point representing the smallest possible difference in scoring within an individual component. These margins were selected in advance to evaluate whether any differences between the two methods remained within clinically acceptable limits. Bootstrap-based 95% confidence intervals for the mean differences between semi-automated and manual PAR and ABO eCRE scores were calculated using 5000 bootstrap resamples. Bootstrapping was performed on the paired model-level score differences, separately for initial and final models. Descriptive tabular and graphical summaries were generated for total scores and subsection to compare automated and manual assessments and to complement the formal noninferiority framework.

## Results

### Participant flow and baseline data

Participant flow is reported in the STROBE study flow chart (Supplementary Figure S1). This study included 50 patients (30 female (60%) and 20 male (40%) with a mean age of 25.32 years (SD 11.30) at the initial scan who met the eligibility criteria and for whom both initial and final models were available. A total of 100 digital models (50 initial and 50 final models) were included in the analysis. The initial models showed a broad distribution of deviation from ideal occlusion, with a median PAR score of 25.0 points [IQR 17.0–33.75] and a median ABO eCRE score of 57.5 points [IQR 48.25–66.0] (Table 1; Supplementary Figure S2).

**Table 1:** Baseline demographic and model characteristics of the study sample.

Characteristic	Overall sample	Format/unit
Patients	50	*n*
Digital models	100	*n*
Initial models	50	*n*
Final models	50	*n*
Age at initial model	25.32 ± 11.30	Years, mean ± SD
Female sex	30 (60%)	*n* (%)
Male sex	20 (40%)	*n* (%)
Initial PAR score	25.0 [17.0–33.75]	Median [IQR]
Initial ABO eCRE score	57.5 [48.25–66.0]	Median [IQR]

ABO eCRE, American Board of Orthodontics electronic Cast-Radiograph Evaluation; PAR, Peer Assessment Rating index.

Values are presented as number and percentage, mean ± standard deviation, and median with interquartile range. Age refers to the time point of the initial model. Initial PAR and ABO eCRE scores describe the degree of deviation from ideal occlusion in the pretreatment models.

### The majority of the differences between automated and manual placement were within the ideal and moderate ranges across tooth types

**Figure 2:**
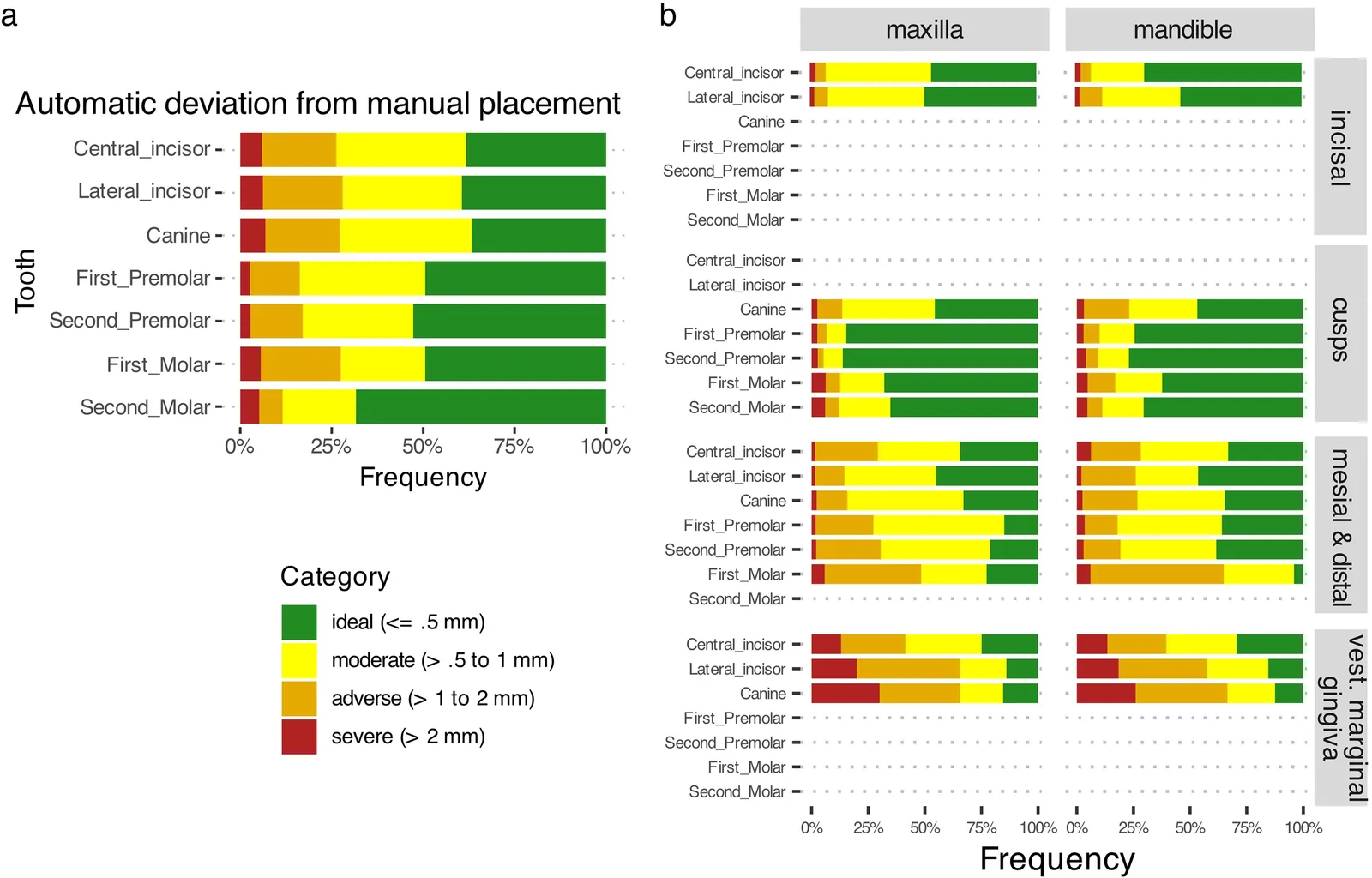
The frequency of deviation in placement of dental landmarks between the automated and manual methods was predominantly in the ‘ideal’ and ‘moderate’ categories across all tooth types. The possible deviation categories are ‘ideal’, ‘moderate’, ‘adverse’, ‘severe’. (a) Frequency of automatic dental landmark deviation categories from manual reference points in 100 patient models, according to tooth group. (b) The frequency of deviation categories of automatic landmarks from manual landmarks (incisal, cusp, mesial, distal and vestibular marginal gingiva), according to tooth group and separately for maxilla or mandible.

Across the seven tooth groups, mean deviations ranged from 0.54 mm to 0.83 mm. The smallest deviations were observed for second molars (mean = 0.54 mm; 95% CI 0.49–0.59 mm) and second premolars (mean = 0.59 mm; 95% CI 0.56–0.62 mm), whereas slightly larger deviations occurred for central incisors (mean = 0.80 mm; 95% CI 0.77–0.83 mm), lateral incisors (mean = 0.81 mm; 95% CI 0.78–0.84 mm), and canines (mean = 0.83 mm; 95% CI 0.80–0.85 mm). Overall, most deviations across tooth types fell within the ideal (≤0.5 mm) or moderate (>0.5–1 mm) categories. Adverse (>1–2 mm) and severe (>2 mm) deviations occurred less frequently (Fig. 2a; Supplementary Table II).

When stratified by jaw, tooth group, and landmark, the cusps showed the smallest deviations, ranging from 0.31 to 0.62 mm in the maxilla and 0.43–0.70 mm in the mandible. Incisal landmarks demonstrated slightly larger deviations (maxilla: 0.57–0.58 mm; mandible: 0.48–0.58 mm). Mesial and distal contact points showed intermediate deviations, ranging from 0.63 to 0.99 mm in the maxilla and 0.71–1.26 mm in the mandible. The largest deviations were observed for landmarks located at the vestibular marginal gingiva, ranging from 1.10 to 1.52 mm in the maxilla and 1.03–1.45 mm in the mandible. Overall, similar deviation patterns were observed between the maxillary and mandibular dentitions (Fig. 2b, Supplementary Table II). Signed coordinate differences along the X-, Y- and Z-axes were centred close to zero, indicating no relevant global directional bias. Bland-Altman shows that the error deltas seem to have certain pattern given the coordinate. Though there is no linear dependency and therefore a systematic bias is not shown. For initial models, bias values were 0.02 mm for X, 0.08 mm for Y and 0.02 mm for Z, with limits of agreement ranging from −1.16 to 1.32 mm. For final models, bias values were −0.01 mm for X, 0.02 mm for Y and 0.04 mm for Z, with limits of agreement ranging from −1.23 to 1.28 mm. No consistent axis-specific displacement pattern was observed (Supplementary Figure S3).

### Comparable landmark placement was observed between initial and final models across tooth types and landmark categories

**Figure 3:**
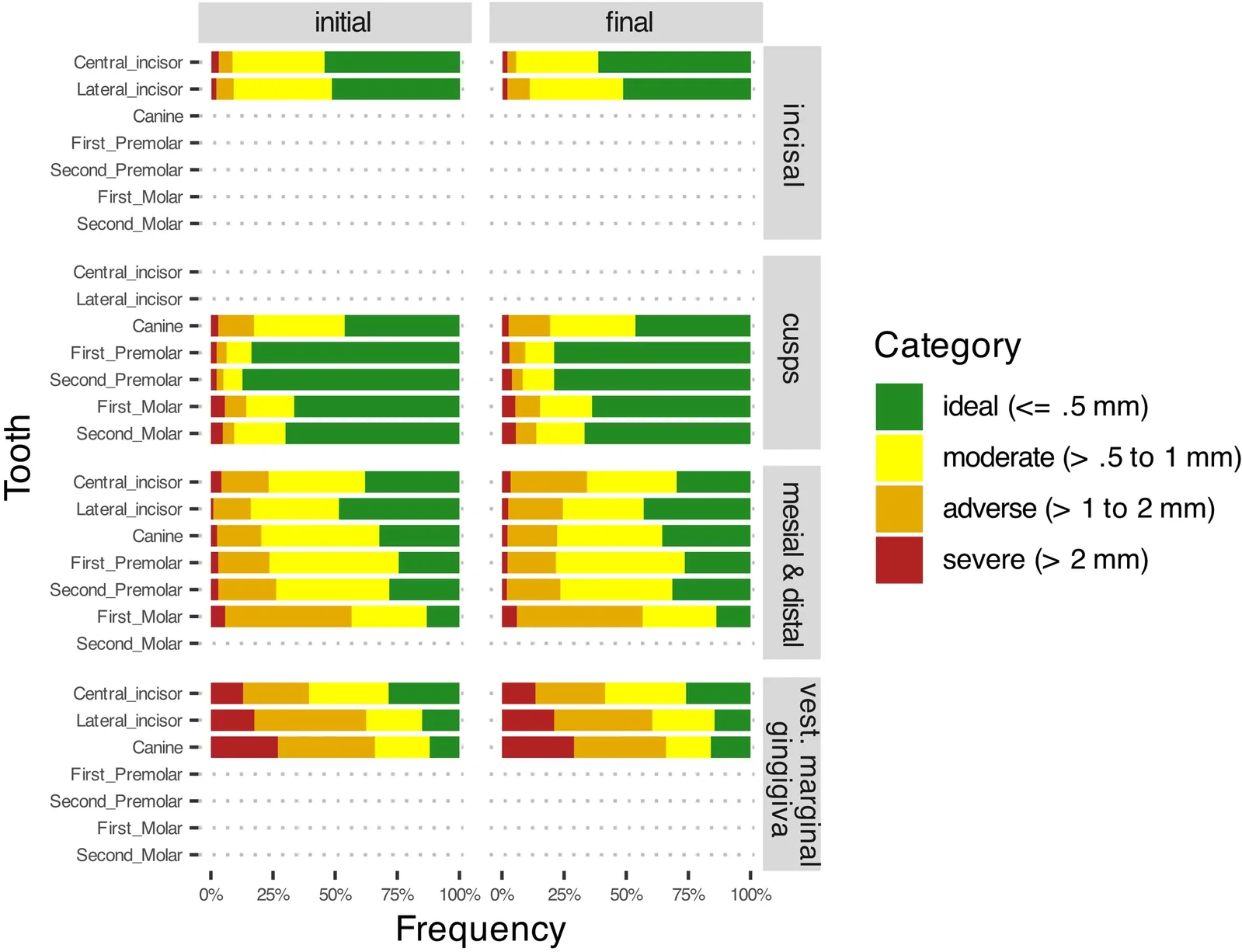
The frequency of discrepancies between automatic and manual dental landmarks (incisal, cusp, mesial, distal, and vestibular marginal gingiva) was analysed by tooth group and for the initial and final model separately. There were no clear differences in the frequency of landmark discrepancies when comparing the initial and final models. Vestibular marginal gingiva showed the worst performance in both types of models.

Across all tooth groups and landmark categories, mean landmark deviations ranged from 0.33–1.47 mm in initial models and 0.40–1.49 mm in final models. Comparison of landmark differences between initial and final models showed no statistically significant differences after correction for multiple testing (Fig. 3, Supplementary Table III).

### Intra- and interrater reliability

Intrarater reliability was high for both automated and manual landmark placement. The automated workflow showed excellent ICC values across all dimensions (ICC = 0.998–0.999), with median coordinate differences of 0.00 mm. Manual placement showed excellent reliability in the X- and Y-dimensions (ICC = 0.999 and 0.995), while the Z-dimension showed lower but still good reliability (ICC = 0.899; 95% CI 0.886–0.910) (Supplementary Table IV). Interrater reliability of manual placement was excellent across all dimensions (ICC = 0.992–0.999), with low median differences of 0.08–0.10 mm (Supplementary Table V).

### Semi-automated and completely manual ABO-eCRE scoring showed small mean differences, but noninferiority was subsection-dependent

**Figure 4:**
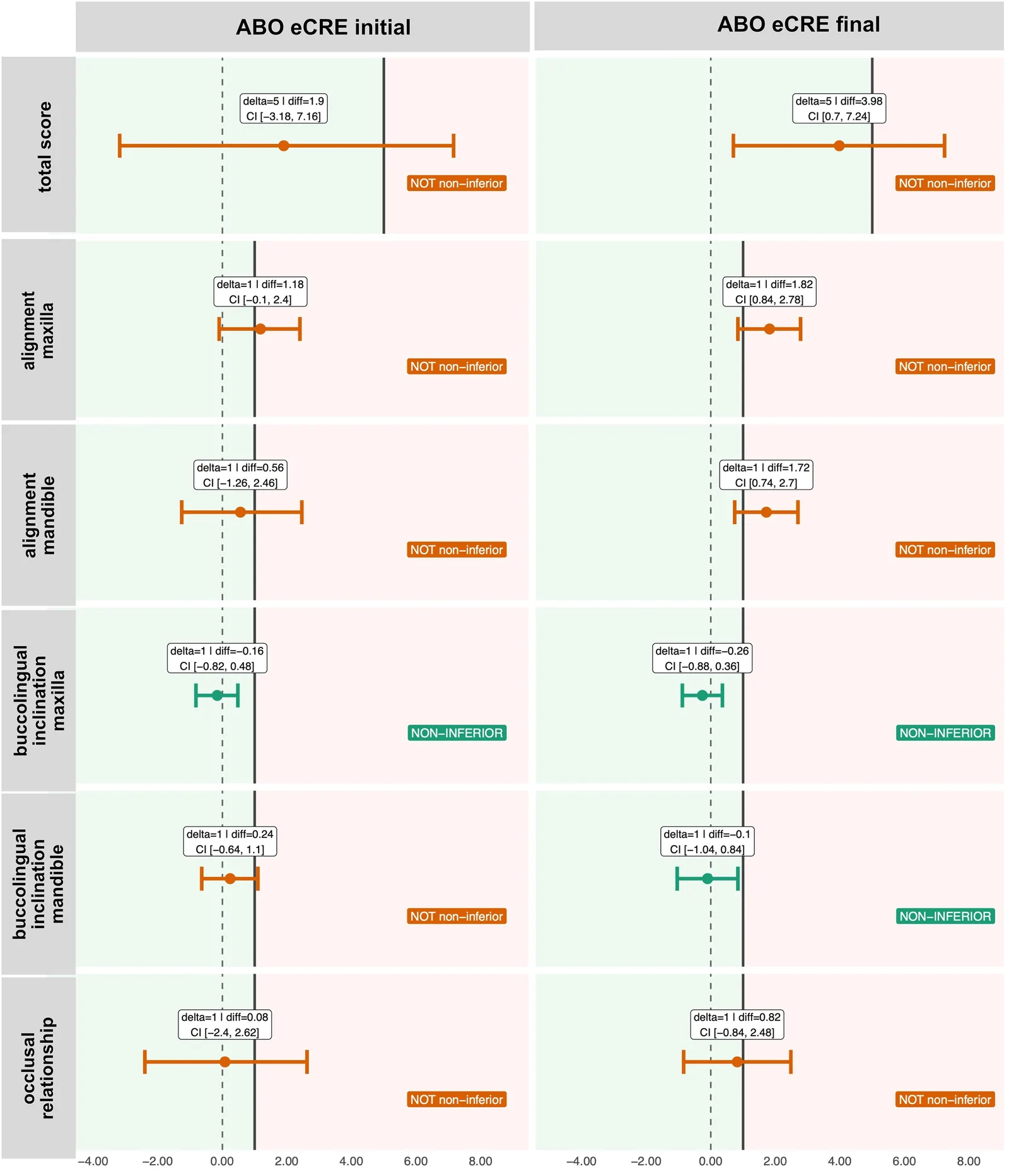
A noninferiority analysis was performed using a semi-automated and full manual ABO-eCRE scoring evaluation by model (initial, final), total score and subsection (alignment maxilla and mandible, buccolingual inclination maxilla and mandible and occlusal relationship). The mean difference between automated and manual scoring is shown with a bootstrap confidence interval (CI).

The total ABO-eCRE score showed that the mean difference between semi-automated and manual scoring was 1.9 points (95% CI −3.18 to 7.16) in the initial models and delta = 3.98 points (95% CI 0.70 to 7.24) in the final models. As the confidence intervals exceeded the predefined margin of 5 points, noninferiority could not be demonstrated for the total ABO eCRE score. The largest mean differences were observed for the alignment subsections in the initial and final models. In contrast, buccal occlusion in the maxilla and mandible showed smaller mean differences and met the predefined noninferiority criterion. For the remaining component, overjet, mean score differences were small, but the confidence intervals exceeded the predefined noninferiority margin in both model stages (initial 0.08, 95% CI −2.40 to 2.62; final 0.82, 95% CI −0.84 to 2.48) (Fig. 4).

### Noninferiority of semi-automated PAR scoring was demonstrated for final models

**Figure 5:**
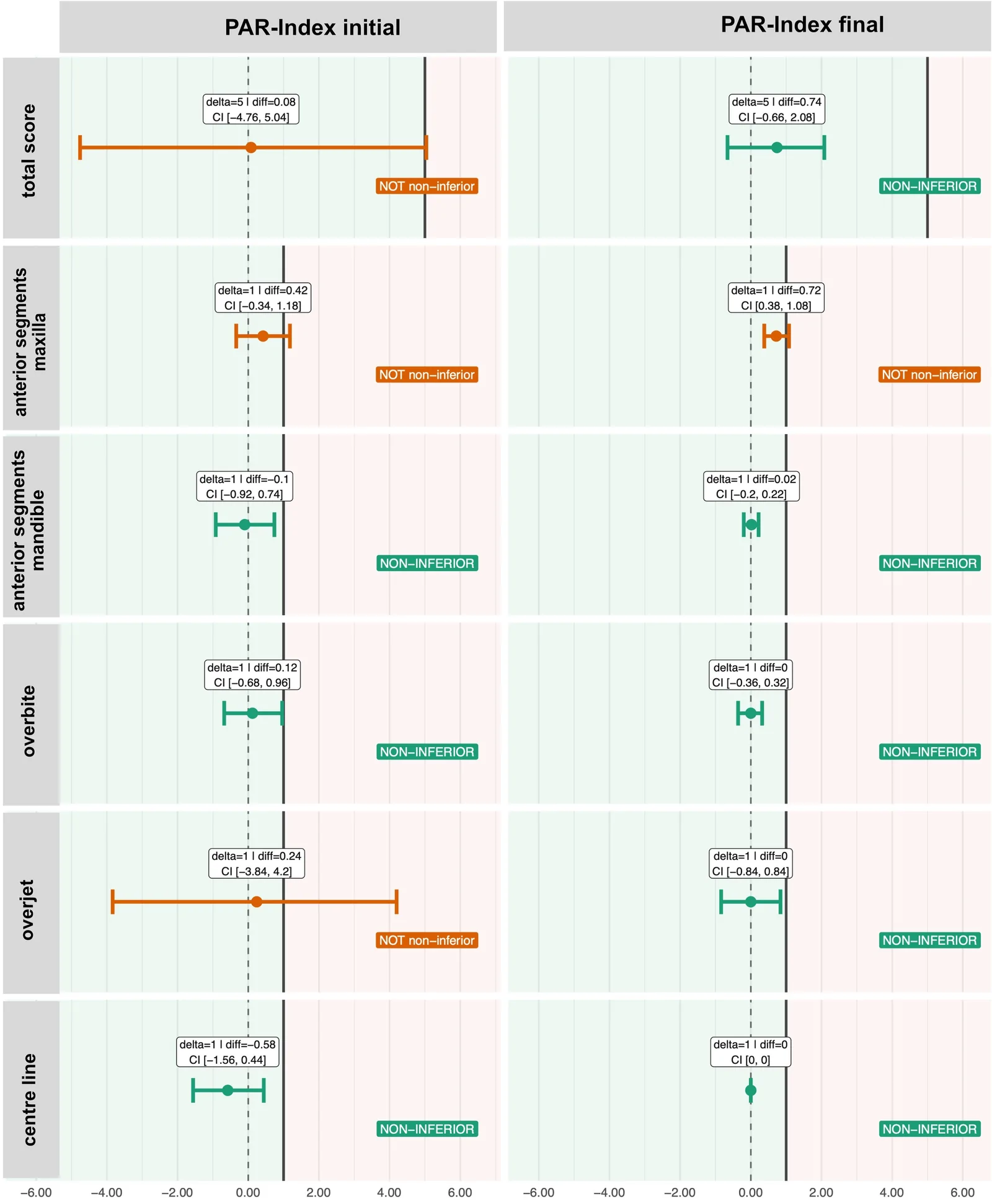
A noninferiority analysis was performed using a semi-automated and full manual PAR-index scoring evaluation by model (initial, final), total score and subsection (anterior segment in maxilla and mandible, overbite, overjet and centre line). The mean difference between automated and manual scoring is shown with a bootstrap confidence interval (CI).

The total PAR score showed a mean difference of −0.08 points (95% CI −4.76 to 5.04) between semi-automated and manual scoring in the initial models, and of 0.74 points (95% CI −0.66 to 2.08) in the final models. The noninferiority criterion was met for the total PAR score in the final models, whereas it could not be demonstrated for the initial models due to the upper confidence interval exceeding the predefined margin. Across the PAR subsections, anterior segments in the mandible, overbite, centre line, and overjet met the predefined noninferiority margin of 1 point in both model stages. In contrast, anterior segments in the maxilla and overjet in the initial models exceeded the predefined margin and therefore did not meet the noninferiority criterion (Fig. 5).

## Discussion

Despite the availability of digital dental models and software-assisted measurements, orthodontic indices for quality assurance are still largely calculated using manual landmark detection, which is a time-consuming process. Automated landmark detection is therefore a promising approach that could reduce the workload of evaluators and enable a standardized assessment process. In this study, landmarks were defined manually by an experienced rater and verified by a second expert. These landmarks were then used as the reference standard and ground truth. In automated landmark detection, expert annotations are generally considered the most accurate representation of reality. The study sample represented consecutive adolescent and adult orthodontic patients from routine clinical practice and sufficient digital scan quality. This age distribution is consistent with the increasing demand for adult orthodontic treatment and reported changes in orthognathic treatment populations over time [15–18]. These patients require restorative treatment more frequently and are more likely to show signs of occlusal wear, gum recession and alveolar bone loss.

Deviations of 1–2 mm in the identification of cephalometric landmarks are generally considered to be within the limits of acceptability for clinical purposes [19–21]. While thresholds derived from two-dimensional radiographic measurements cannot be applied directly to three-dimensional identification, they can nevertheless serve as a useful guide.

Occlusal thresholds primarily refer to calculated distances, such as tooth width, and deviations of ∼0.5 mm are generally considered acceptable [2, 22]. Orthodontic quality indices can also serve as a useful benchmark. For example, the ABO-CRE penalizes deviations of 0.5–1.0 mm between two landmarks by 1 point, whereas deviations of 2 mm or more incur two penalty points. In contrast, the PAR only awards penalty points for deviations of 1.0 mm or more [6, 10].

Based on these considerations, we have defined the following thresholds for the automated identification of dental landmarks: ≤0.5 mm (ideal); >0.5–1.0 mm (moderate), which is clinically acceptable [23]; >1.0 mm (adverse), which is a potentially relevant deviation in an orthodontic context as both indices assign penalty points for deviations greater than this value. Deviations of more than 2 mm were classified as severe as they exceed the generally accepted clinical tolerance threshold for landmarks.

Clearly defined, transparent and standardized thresholds could therefore be particularly valuable in the future for the development and validation of digital applications and automated landmark recognition systems in orthodontics.

The accuracy of automated landmark detection on digital dental models varies depending on the type of landmark [12]. Deviations specific to each tooth group remained within an acceptable range of on average across the categories defined in our study. The greatest differences between automated and manual landmark placement were observed in the anterior teeth and canines. These differences can be explained by the anatomical characteristics of these tooth types. The highest level of agreement was found for cusp landmarks, since cusp tips represent clearly defined anatomical structures that can be reliably identified. Previous studies have also reported the effectiveness of automated approaches in detecting cusp landmarks [24]. Mesial and distal contact points showed slightly greater discrepancies, particularly for the first molar. This may be due to the difficulty in determining the maximum mesiodistal crown dimension. The comparatively rounded morphology of these areas, combined with the limited accessibility of the proximal contact area, could make precise landmark localization challenging [24]. Vestibular marginal gingival landmarks showed the largest deviations from manual expert placement, which is plausible given their less clearly defined anatomical location. In line with previous reports, cervical landmarks were shown to exhibit higher error rates than occlusal landmarks [23]. These landmarks should therefore be considered critical in automated workflows. Although they were designed to reflect the full semi-automated workflow, they should be carefully verified after automated placement. Where necessary, they should be placed manually, particularly when used for index-based assessments. In general, the absence of clearly defined anatomical characteristics can affect the accuracy of manual and algorithmic landmark placement. If such landmarks are used extensively in automated analyses or index-based assessments, this should be considered, since small individual deviations can build up and potentially impact the result. Similar patterns of deviation were observed within each tooth class (anterior teeth, canines, premolars and molars) regarding tooth- and landmark-specific deviations. Consequently, it can be assumed that measurement deviation depends more heavily on the anatomy of the tooth class than on the individual tooth. The coordinate-wise analysis was performed across all landmarks and separately for initial and final models to assess whether the automated workflow showed a global directional displacement within the oriented model coordinate system. The signed differences were centred close to zero in the X-, Y- and Z-axes, indicating no relevant overall shift in the transverse, vertical or sagittal direction. The deviations appear to be driven by landmark-specific variability, particularly for anatomically less clearly defined landmarks such as vestibular marginal gingival points. The excellent intra- and interrater reliability supports the robustness of the manual landmark placement used as the reference standard [25]. Similarly, manual landmark placement demonstrated excellent reliability in the horizontal dimension, reflecting the ability of experienced users to consistently identify anatomical reference points on digital models. The slightly lower reliability observed in the *z*-axis during manual placement can be attributed to the greater difficulty in precisely defining the sagittal dimension. This is because there are no clear anatomical boundaries here, particularly for the mesial and distal or gingival landmarks [24]. However, the high level of agreement between the raters suggests that this variability can be minimized by standardizing the definition of the orientation points. The perfect ICCs observed with the automated approach suggest that landmark detection is extremely stable and reproducible, regardless of the segmentation process. It should be noted that careful segmentation of the model was performed in both cases [26]. Overall, these findings suggest that the differences observed between automated and manual landmark placement are unlikely to be due to the limited reproducibility of the reference standard.

Although learning-based approaches are increasingly being investigated [26, 27], the performance of nonmachine learning-based algorithmic methods for automated landmark detection has received less attention. The quality of the output depends on the quality of the input, which must be precise and high-quality. A variety of software tools are available to support the analysis of digital models with user-defined landmarks. It has been demonstrated that the limitations of these tools are solely due to the landmark detection [7].

For the total ABO eCRE score, a maximum difference of 5 points was defined as *a priori*, based on previous ABO Model Grading System studies in which a 5-point difference in the mean total score was considered clinically meaningful or clinically significant [13, 14]. For the total PAR score, the same 5-point margin was used as a clinically interpretable threshold for method comparison, as larger PAR score changes are generally required to indicate substantial treatment improvement. For individual score components, a 1-point margin was selected because both indices use discrete scoring categories and one point represents the smallest possible component-level score difference.

A comparison of ABO values determined using automated methods (SureSmile, OraMetrix, Richardson, Texas software) or manual, user-defined landmarks on digital models with the manual evaluation of plaster models shows significantly higher values for three criteria: alignment, occlusal contacts, and overbite [28, 29]. According to our study, the semi-automated ABO-eCRE score involves manual evaluation of components such as occlusal contacts and overjet, while alignment is predominantly determined automatically to reduce workload. While the mean differences between the semi-automated and manual assessments were generally within acceptable clinical limits, the confidence intervals for several endpoints exceeded the predefined noninferiority limits. This was particularly evident in the final ABO-eCRE score. Therefore, the noninferiority of the semi-automated ABO-eCRE assessment could not be demonstrated. The clinical implications of this difference are unclear, since the ABO-eCRE was analysed as a continuous quality assessment score and there is no officially validated threshold for altering clinical decisions. Nevertheless, higher semi-automated ABO-eCRE scores may indicate a more conservative assessment in some cases. For PAR, agreement was more favourable, especially in final models, but noninferiority was not consistently supported across all comparisons. Similarly, some component-level analyses exceeded the predefined margin of 1 point, indicating that agreement varied across indices and score components. However, discrepancies were identified when the final models were assessed. This is probably due to the strict scoring rules of the ABO-CRE, whereby deviations of ≥1 mm result in a two-point penalty, whereas larger deviations are not penalized as severely. It has been demonstrated that semi-automated PAR can deliver reliable and valid results in digital orthodontic analysis [3, 29, 30]. The deviation in the total PAR score and in the respective sub-sections was marginal for both models. The smaller deviations compared with the ABO score may be due to the index's lower stringency [31, 32]. Furthermore, the assessment uses significantly fewer landmark points, which reduces measurement error accordingly.

These findings should not be interpreted as evidence of general equivalence between semi-automated and manual scoring. Instead, they suggest that semi-automated scoring may provide similar mean estimates in some settings, but with outcome-dependent uncertainty. This is clinically relevant because even small landmark deviations may influence score components differently, depending on the structure and weighting of the respective index.

Quantifying the quality of care is essential for developing personalized, optimized treatment strategies. This ultimately benefits all patients, as treatment decisions can be made efficiently and objectively based on reliable findings. Our results can therefore be used to improve semi-automated approaches and develop automated solutions. However, it is important to note that the review of results and their application to diagnosis and treatment remains the responsibility of a specialist. Nevertheless, clinicians should carefully review all automatically generated assessments produced as part of semi-automated workflows. This is particularly important in orthodontics, to avoid placing blind reliance on automated applications or analyses.

### Limitations

The retrospective design limited control over patient- and tooth-related characteristics, such as tooth wear, restorations, gingival recession and crown morphology, which may have influenced landmark identification. Although baseline PAR and ABO eCRE scores indicated variable deviation from ideal occlusion, specific malocclusion types and treatment-complexity levels were not analysed separately. The influence of individual clinical characteristics on automated landmark accuracy therefore remains to be investigated.

### Generalisability

As models of insufficient scan quality were excluded, the findings may not fully represent performance under suboptimal clinical conditions. The age distribution reflects current trends towards increasing adult orthodontic demand but limits direct generalisability to younger patients in mixed or early permanent dentition. As the landmark detection algorithm is proprietary, the findings apply to the commercially available OnyxCeph^3^™ workflow as implemented and may not be directly generalizable to other software systems.

## Conclusions

Automated landmark detection generally showed acceptable deviations from expert-defined reference annotations across tooth types and landmark categories, particularly for cusp and incisal landmarks. However, the magnitude and variability of these discrepancies differed between landmark types, with vestibular marginal gingival landmarks showing the greatest discrepancies. Semi-automated PAR and ABO eCRE scoring produced small mean differences compared with manual assessment, though the degree of agreement varied across indices and score components. These findings suggest that, while automated landmark detection can support standardized digital orthodontic analysis and index calculation, it should not be regarded as fully autonomous. Clinical verification remains necessary, particularly for anatomically ambiguous landmarks and score components with greater variability.

## Supplementary Material

cjag063_Supplementary_Data

## Data Availability

The data supporting the findings of this study are available from the corresponding author upon request.
