# Supplementary material for: Comparison of automated algorithm-based versus manual dental landmark placement for model analysis and evaluation of orthodontic quality indices
Source: Eur J Orthod. 2026 Aug 26;48(5):cjag063. doi: 10.1093/ejo/cjag063 (PMC13507319; doi:10.1093/ejo/cjag063)
Supplement: Supplementary file 1 [file cjag063_supplementary_data.docx]

**Comparison of automated algorithm-based versus manual dental landmark placement for model analysis and evaluation of orthodontic quality indices**

**Supplementary Figure 1.** STROBE study flowchart of patient cohort

**Supplementary Figure 2.** Baseline distribution of ABO eCRE and PAR scores in the initial models**.**
Density plots showing the distribution of baseline ABO eCRE and PAR scores for the initial digital models. Boxplots indicate the median and interquartile range. The baseline scores showed a broad distribution, reflecting variability in the degree of deviation from ideal occlusion within the consecutive sample. ABO eCRE, American Board of Orthodontics electronic Cast-Radiograph Evaluation; PAR, Peer Assessment Rating index.

**Supplementary Figure 3.** Density plots illustrate the distribution of deviations for the x-, y-, and z-coordinates of the evaluated landmarks in the initial and final datasets. The red line indicates the median, the blue line the mean, and the green line the median value. Bland–Altman plots show the differences between repeated measurements plotted against the mean raw coordinate values for the x-, y-, and z-directions. The solid line represents the mean bias, whereas the dashed lines indicate the upper and lower limits of agreement. The corresponding bias values and limits of agreement are displayed within each plot. Negative and positive deviations represent directional differences between the compared coordinate datasets.

**Supplementary Table I.** Strobe checklist

|  | Item No | Recommendation | Location where the item is reported and page number |
| --- | --- | --- | --- |
| **Title and abstract** | 1 | (*a*) Indicate the study’s design with a commonly used term in the title or the abstract | Page 1 |
|  |  | (*b*) Provide in the abstract an informative and balanced summary of what was done and what was found | Page 1 |
| Introduction | | |  |
| Background/rationale | 2 | Explain the scientific background and rationale for the investigation being reported | Introduction, page 2 |
| Objectives | 3 | State specific objectives, including any prespecified hypotheses | Introduction, page 2 |
| Methods | | |  |
| Study design | 4 | Present key elements of study design early in the paper | Material and methods, page 3, Figure 1 |
| Setting | 5 | Describe the setting, locations, and relevant dates, including periods of recruitment, exposure, follow-up, and data collection | Material and methods, page 3, Supplementary Figure 1 |
| Participants | 6 | (*a*) Give the eligibility criteria, and the sources and methods of selection of participants. Describe methods of follow-up | Material and methods, page 3 |
|  |  | (*b*) For matched studies, give matching criteria and number of exposed and unexposed | N/A |
| Variables | 7 | Clearly define all outcomes, exposures, predictors, potential confounders, and effect modifiers. Give diagnostic criteria, if applicable | Material and methods, page 5; All outcomes and sources of measurement variability were defined. Confounder adjustment was not applicable because this was a paired within-model method-comparison study without estimation of a causal exposure–outcome association. |
| Data sources/ measurement | 8* | For each variable of interest, give sources of data and details of methods of assessment (measurement). Describe comparability of assessment methods if there is more than one group | Material and methods, page 4 |
| Bias | 9 | Describe any efforts to address potential sources of bias | Material and methods, page 5 |
| Study size | 10 | Explain how the study size was arrived at | Material and methods, page 6 |
| Quantitative variables | 11 | Explain how quantitative variables were handled in the analyses. If applicable, describe which groupings were chosen and why | Material and methods, page 6 |
| Statistical methods | 12 | (*a*) Describe all statistical methods, including those used to control for confounding | Material and methods, page 6-7 |
|  |  | (*b*) Describe any methods used to examine subgroups and interactions | N/A |
|  |  | (*c*) Explain how missing data were addressed | Supplementary Figure 1 |
|  |  | (*d*) If applicable, explain how loss to follow-up was addressed | N/A |
|  |  | (*e*) Describe any sensitivity analyses | N/A |
| Results | | |  |
| Participants | 13* | (a) Report numbers of individuals at each stage of study—eg numbers potentially eligible, examined for eligibility, confirmed eligible, included in the study, completing follow-up, and analysed | Results, page 7-8; Supplementary Figure 1 |
|  |  | (b) Give reasons for non-participation at each stage | Supplementary Figure 1 |
|  |  | (c) Consider use of a flow diagram | Supplementary Figure 1 |
| Descriptive data | 14* | (a) Give characteristics of study participants (eg demographic, clinical, social) and information on exposures and potential confounders | Results, page 7; Table I |
|  |  | (b) Indicate number of participants with missing data for each variable of interest | N/A |
|  |  | (c) Summarise follow-up time (eg, average and total amount) | N/A |
| Outcome data | 15* | Report numbers of outcome events or summary measures over time | Results, page 7-8 |
| Main results | 16 | (*a*) Give unadjusted estimates and, if applicable, confounder-adjusted estimates and their precision (eg, 95% confidence interval). Make clear which confounders were adjusted for and why they were included | Results, page 7-8 |
|  |  | (*b*) Report category boundaries when continuous variables were categorized | N/A |
|  |  | (*c*) If relevant, consider translating estimates of relative risk into absolute risk for a meaningful time period | N/A |
| Other analyses | 17 | Report other analyses done—eg analyses of subgroups and interactions, and sensitivity analyses | Results 8 |
| Discussion | | |  |
| Key results | 18 | Summarise key results with reference to study objectives | Discussion, page 8-9 |
| Limitations | 19 | Discuss limitations of the study, considering sources of potential bias or imprecision. Discuss both direction and magnitude of any potential bias | Discussion, page 13 |
| Interpretation | 20 | Give a cautious overall interpretation of results considering objectives, limitations, multiplicity of analyses, results from similar studies, and other relevant evidence | Discussion, page 9-10 |
| Generalisability | 21 | Discuss the generalisability (external validity) of the study results | Discussion, page 13 |
| Other information | | |  |
| Funding | 22 | Give the source of funding and the role of the funders for the present study and, if applicable, for the original study on which the present article is based | Page 14 |

*Give information separately for exposed and unexposed groups.

**Note:** An Explanation and Elaboration article discusses each checklist item and gives methodological background and published examples of transparent reporting. The STROBE checklist is best used in conjunction with this article (freely available on the Web sites of PLoS Medicine at http://www.plosmedicine.org/, Annals of Internal Medicine at http://www.annals.org/, and Epidemiology at http://www.epidem.com/). Information on the STROBE Initiative is available at <http://www.strobe-statement.org>.

**Supplementary Table II.** Descriptive statistics of dental landmark deviations between automated and manual placement. (I) summarises deviations by tooth group. (II) provides detailed deviations of the tooth groups by jaw and landmark type. Values are presented as mean, standard deviation (SD), median, interquartile range (Q1–Q3), minimum (Min), maximum (Max), and frequency distribution across deviation categories (ideal ≤0.5 mm, moderate >0.5–1 mm, adverse >1–2 mm, severe >2 mm). Results are stratified by tooth group, jaw (maxilla and mandible), and landmark type (cusp, incisal, mesial/distal contact points [m&d], and vestibular marginal gingiva [vMG]). Confidence intervals (95% CI) are provided for mean deviations.

**Supplementary Table III.** Descriptive statistics of tooth group deviations stratified by model stage and landmark type. Values are presented as mean, standard deviation (SD), median, interquartile range (Q1–Q3), minimum (Min), maximum (Max), and frequency distribution across deviation categories (ideal ≤0.5 mm, moderate >0.5–1 mm, adverse >1–2 mm, severe >2 mm). Results are stratified by tooth group, model type (initial and final), and landmark type (cusp, incisal, mesial/distal contact points [m&d], and vestibular marginal gingiva [vMG]). Confidence intervals (95% CI) are provided for mean deviations.

**Supplementary Table IV.** Intrarater reliability of automated and manual landmark placement across spatial dimensions (X, Y, Z). Intraclass correlation coefficients (ICC) with corresponding 95% confidence intervals (CI) are reported for each dimension. Mean differences (mm) and standard deviations (SD) describe the accuracy between repeated measurements.

| Rater | Dimension | ICC | Lower CI | Upper_CI | Median | Mean_Diff (mm) | SD |
| --- | --- | --- | --- | --- | --- | --- | --- |
| Automated | X | 0.9998310 | 0.9998074 | 0.9998516 | 0.00 | 0 | 0,14 |
|  | Y | 0.9983236 | 0.9980903 | 0.9985284 | 0.00 | -0,01 | 0,14 |
|  | Z | 0.9997355 | 0.9996986 | 0.9997679 | 0.00 | 0,01 | 0,16 |
| Manual | X | 0.9986921 | 0.9985201 | 0.9988434 | 0.02 | 0,04 | 0,39 |
|  | Y | 0.9950804 | 0.9944610 | 0.9956307 | 0.00 | 0,01 | 0,23 |
|  | Z | 0.8986732 | 0.8857906 | 0.9100954 | 0.00 | -0,42 | 3,21 |

**Supplementary Table V.** Interrater reliability of manual landmark placement across spatial dimensions (X, Y, Z). Intraclass correlation coefficients (ICC) with corresponding 95% confidence intervals (CI) are reported for each dimension. Mean differences (mm) and standard deviations (SD) describe the accuracy between the two raters.

| Dimension | ICC | Lower_CI | Upper_CI | Median | Mean_Diff (mm) | SD |
| --- | --- | --- | --- | --- | --- | --- |
| X | 0.9979369 | 0.9975228 | 0.9982819 | 0.10 | 0.19 | 0.42 |
| Y | 0.9918323 | 0.9900242 | 0.9932929 | 0.08 | 0.17 | 0.28 |
| Z | 0.9986327 | 0.9983582 | 0.9988613 | 0.10 | 0.16 | 0.33 |
